# Preliminary Study of Acute Changes in Emotion Processing in Trauma Survivors with PTSD Symptoms

**DOI:** 10.1371/journal.pone.0159065

**Published:** 2016-07-14

**Authors:** Xin Wang, Hong Xie, Andrew S. Cotton, Elizabeth R. Duval, Marijo B. Tamburrino, Kristopher R. Brickman, Jon D. Elhai, S. Shaun Ho, Samuel A. McLean, Eric J. Ferguson, Israel Liberzon

**Affiliations:** 1 Department of Psychiatry, University of Toledo, Toledo, Ohio, United States of America; 2 Department of Neurosciences, University of Toledo, Toledo, Ohio, United States of America; 3 Department of Psychiatry, University of Michigan, Ann Arbor, Michigan, United States of America; 4 Department of Emergency Medicine, University of Toledo, Toledo, Ohio, United States of America; 5 Department of Psychology, University of Toledo, Toledo, Ohio, United States of America; 6 Department of Anesthesiology, University of North Carolina at Chapel Hill, Chapel Hill, North Carolina, United States of America; 7 Department of Trauma service, ProMedica Toledo Hospital, Toledo, Ohio, United States of America; Central Institute of Mental Health, GERMANY

## Abstract

Accumulating evidence suggests traumatic experience can rapidly alter brain activation associated with emotion processing. However, little is known about acute changes in emotion neurocircuits that underlie PTSD symptom development. To examine acute alterations in emotion circuit activation and structure that may be linked to PTSD symptoms, thirty-eight subjects performed a task of appraisal of emotional faces as their brains were functionally and structurally studied with MRI at both two weeks and three months after motor vehicle collision (MVC). As determined by symptoms reported in the PTSD Checklist at three months, sixteen survivors developed probable PTSD, whereas the remaining 22 did not meet criteria for PTSD diagnosis (non-PTSD). The probable PTSD group had greater activation than the non-PTSD group in dorsal and ventral medial prefrontal cortex (dmPFC and vmPFC) while appraising fearful faces within two weeks after MVC and in left insular cortex (IC) three months after MVC. dmPFC activation at two weeks significantly positively correlated with PTSD symptom severity at two weeks (R = 0.462, P = 0.006) and three months (R = 0.418, p = 0.012). Changes over time in dmPFC activation and in PTSD symptom severity were also significantly positively correlated in the probable PTSD group (R = 0.641, P = 0.018). A significant time by group interaction was found for volume changes in left superior frontal gyrus (SFG, F = 6.048, p = 0.019) that partially overlapped dmPFC active region. Between two weeks and three months, left SFG volume decreased in probable PTSD survivors. These findings identify alterations in frontal cortical activity and structure during the early post-trauma period that appear to be associated with development of PTSD symptoms.

## Introduction

Millions of Americans experience trauma at some point in life [1]. In some but not all trauma survivors, trauma exposure leads to development of post-traumatic stress disorder (PTSD) [2, 3]. PTSD is found in about 2.6–6.8% of the United States population [4, 5] and is characterized by persisting symptoms including re-experience of the trauma, avoidance of trauma reminders, and hyperarousal for a month or longer after the trauma [6]. It is important to understand how PTSD develops to predict and prevent its occurrence.

PTSD has been linked to deficits in processing and regulation of negative emotions, suggesting potential abnormalities in brain regions involved in emotion processing and regulation [6, 7]. Existing work suggests that compared to non-PTSD samples, chronic PTSD is associated with greater activity in emotion processing regions, including amygdala and insular cortex (IC), and less activity in emotion regulatory regions, including dorsolateral prefrontal (dlPFC), medial prefrontal (mPFC), and anterior cingulate (ACC) cortex [8, 9]. Reduced gray matter volume, density, and thickness in hippocampus, prefrontal cortices (PFC), and ACC have also been associated with chronic PTSD symptoms [10–12]. Although these functional and structural changes have been documented in chronic PTSD, little is known about the temporal development of brain changes that lead to PTSD symptoms. Recognition of early brain changes that precede chronic PTSD can provide clues to mechanisms of PTSD development vs. uneventful post-trauma recovery [13, 14]. Surprisingly, early post-trauma changes in brain function and structure have rarely been studied in trauma survivors who subsequently develop PTSD [13].

Recent reports suggest that trauma exposure can trigger rapid brain changes within days of the event [15–21]. From two days to one month after trauma, activation to trauma-related stimuli is greater in PFC and right IC, but less in amygdala and hippocampus of trauma survivors compared to non-trauma exposed controls [17–19]. Traumatic experiences acutely alter functional coupling between amygdala and IC or hippocampus during processing of trauma-related stimuli [17] and between frontal-limbic-striatal and default mode network regions during rest [16, 20]. Some early changes in functional connectivity may persist for two years following trauma [20]. Contributions of these acute post trauma changes to PTSD development have received little attention. Limited data suggest inter-hemispheric functional connectivity and white matter integrity differ in the days after trauma in motor vehicle collision (MVC) survivors who do versus do not develop PTSD one to six months after MVC [22, 23]. Progressive decreases in white matter integrity in left IC from two days to six months after trauma have been reported in survivors who developed PTSD [22]. A decrease in responses to trauma-related stimuli in right PFC and other regulatory regions from two months to two years following a mining accident in patients who developed PTSD further suggests that progressive changes in emotion regulatory circuits might be involved in PTSD development [24]. These clues aside, early and progressive post-trauma changes in emotional circuitry and their potential to predict PTSD development require further study.

The present investigation examined brain activation patterns and structure of emotional circuits within two weeks and at three months after MVC to assess relationships between early and progressive brain changes and development of PTSD symptoms.

## Materials and Methods

### Subjects

Adults, 18–60 years old, were recruited from hospital emergency departments (EDs) within 48 hours after MVC. Survivors were excluded if they were pregnant, under the influence of alcohol or drugs at the time of MVC, or had major injuries (i.e., Abbreviated Injury Scale [AIS] score >2), moderate to severe traumatic brain injury, major medical illness affecting general health, or conditions that precluded assessment or MRI procedures. An initial sample of 72 survivors completed written informed consent in the EDs and participated in the initial MRI scan within 2 weeks after MVC. Forty-two of the initial 72 subjects returned for a 3 month follow-up MRI scan. Attrition of survivors was due to self-withdrawal, loss of contact, changes in physical condition, or motion artifacts in scans. Thirty-eight survivors with useable functional MRI (fMRI) data at both time points were included in final analyses. Thirty-two of them answered a question about damage to their vehicle, and 29 (91%) rated the damage to be moderate to severe, i.e., no longer drivable or totally destroyed. Survivors had physical injuries that were considered by first responders as threatening to well-being and requiring immediate medical attention in EDs. The University of Toledo Institutional Review Board approved this study.

### Symptom assessments

To assess post-traumatic stress symptoms, all survivors completed the PTSD Checklist-Stressor Specific Version (PCL), in which the MVC was specified as the index trauma [25]. Survivors were grouped into probable PTSD or non-PTSD groups based on PTSD symptoms at 3 months after MVC. Based on PCL responses, the probable PTSD group included survivors who met the DSM-IV-TR criteria for PTSD (i.e., 1 re-experiencing, 3 avoidance/numbing, and 2 hyperarousal symptoms), or partial PTSD (i.e., 1 re-experiencing, and either 3 avoidance/numbing or 2 hyperarousal symptoms) [26, 27]. Survivors with partial PTSD were included in the probable PTSD group because partial PTSD with impairment of social function often requires clinical intervention [27]. The non-PTSD group included survivors who did not meet the above full or partial PTSD criteria. Acute MVC related pain was evaluated using the Numeric Pain Scale (NPS) at ED admission. A Mini-International Neuropsychiatric Interview (M.I.N.I. Version 6.0.0) assessed comorbid psychiatric conditions at three months [28]. A mild traumatic brain injury (mTBI) diagnosis was established using American Congress of Rehabilitation Medicine criteria by reviewing ED medical records and self-report questionnaires [29]. Perceived social support received by survivors was evaluated using a Multidimensional Scale of Perceived Social Support (MSPSS) that was shortened to 4 questions by not distinguishing whether support sources were a special person, family, or friend [30]. Psychotropic medication use was identified from ED medical records and self-report surveys.

### MRI data acquisition

Survivors were scanned using a 3 T General Electric Signa HDx MRI scanner. fMRI images were acquired with a T2*-weighted, Echo Planar Imaging pulse sequence [gradient echo pulse sequence, repetition time (TR) = 2000ms, echo time (TE) = 30ms, flip angle (FA) = 90°, field of view (FOV) = 240mm, matrix = 64×64, slice thickness = 3.5mm with no gaps, 34 axial interleaved slices to cover the whole brain, and with 225 phases obtained in each run]. A T1-weighted scan was obtained for overlay of the fMRI data using a gradient recall echo (GRE) sequence (2 excitations, TR = 250ms, TE = 3.6ms, FA = 90°, FOV = 240mm, matrix = 64×64, slice thickness = 3.5mm, 34 axial slices to cover the whole brain). A further higher resolution T1-weighted structural MRI image was also obtained with a 3-D Volume Inversion Recovery Fast Spoiled Gradient Recall Echo (IR-FSPGR) protocol (TR = 7.9ms, TE = 3ms, TI = 650ms, FOV = 256mm, matrix = 256×256, slice thickness = 1mm, voxel dimensions = 1×1×1mm, 164 contiguous axial slices) [31]. MRI brain images were reviewed by a radiologist and no qualitative clinical abnormalities were detected.

### fMRI paradigm and analysis

The Shifted-attention Emotion Appraisal Task (SEAT) described in our previous works, was used to examine activation in brain regions associated with implicit emotional processing, attention modulation of emotion, and cognitive appraisal [32, 33]. In brief, subjects viewed a grayscale composite picture depicting an emotional face (angry, fearful, or neutral faces selected from the Face Suite published by Paul Ekman Group LLC http://www.paulekman.com/product/face-suite-combo/) superimposed on an indoor or outdoor scene. On each trial, survivors determined whether: (a) the face was male or female (Male/Female) to probe implicit emotional processing, (b) the scene was indoor or outdoor (Indoor/Outdoor) to probe attention modulation of emotion, or (c) whether they liked or disliked the face (Like/Dislike) to probe modulation of emotion by cognitive appraisal. Each trial lasted 1.5 seconds, and inter-trial intervals were randomized from 3 to 8 seconds. Fourteen trials for each trial type and 10 trials each of the control condition (uncompounded face or place) were divided into 3 runs of about 7 minutes per run in each session. To control for practice effects and habituation, no compound face/place picture was repeated for the two sessions. Paradigms were programmed with E-Prime (PST, Inc., Pittsburgh, PA). Stimuli were presented with an MRI specialized visual goggle presentation system (NordicNeuroLab), and responses were recorded via a fiber optic button response system (Psychology SoftwareTools, Inc.). Previous SEAT studies in healthy subjects have shown activation increases in left dorsal mPFC (dmPFC) and IC during cognitive appraisal of emotional facial expressions [32]. We specifically used the appraisal trials to probe emotion modulation following MVC. Comparing appraisal of threat (fearful or angry) faces versus neutral faces also permitted study of deficits in negative emotion regulation, often reported in patients with PTSD [34].

All fMRI data were processed using FSL (version 5.2.0, FMRIB, Oxford, UK) [35]. fMRI images underwent removal of the initial 4 volumes, skull-striping, slice timing correction, motion correction, high-pass temporal filtering (128 seconds), smoothing using a Gaussian kernel of full width at half maximum (FWHM) of 5 mm, and linear registration to standard space [12 degrees of freedom (DOFs)] using each subject’s low and high resolution anatomic images as intermediate steps (9 and 12 DOFs, respectively). The E-Prime time series was convolved with a double-gamma hemodynamic response function. Activation was calculated as contrasts between trial types using fixed effects and then averaged across runs in the same time point within each subject. In the voxel-based whole brain analyses, the activation during appraisal was compared between probable PTSD and non-PTSD groups at 2 weeks after MVC using FLAME-1 (FMRIB's Local Analysis of Mixed Effects) with a general linear model (GLM) of two sample t-tests, controlling for age and gender. Appraisal of fearful or angry faces versus neutral faces was also compared independently at both time points between groups controlling for age and gender, to examine early alterations of activation associated with appraisal of negative emotional stimuli. A grey matter mask created from the 25% probability map of the Harvard-Oxford Cortical and Subcortical Structural Atlases in FSL package was applied in Pre-threshold masking. Whole brain family-wise error rate (FWE) correction was applied on z-statistic images at a voxel threshold of z>2.3 (p<0.01) or above and a cluster significance threshold of p<0.05 [36]. In the post hoc regions of interest (ROI) analysis, the average percent change in contrast of parameter estimates (COPEs) was extracted from a 4 millimeter diameter sphere centered at the peak voxel identified in the above whole brain analyses at each time point using FSL/featquery to further analyze the task-related activations in SPSS (version 21). To explore potential effects of acute pain on the identified activation differences, the extracted COPEs at each time point were compared between groups using univariate ANOVA with acute pain scores as a covariate in addition to factors of age, gender and group. Furthermore, repeated measures analyses of variance (RM-ANOVA) were used to analyze effects of time by group interactions on extracted COPEs. Within each group, measures at the two time points were also compared using RM-ANOVA to identify changes over time.

### Structural MRI analysis

Automated methods to measure cortical volume were performed using FreeSurfer (version 4.5.1) [37]. The automated procedures produced segmentation and parcellation of cortex according to the FreeSurfer Desikan-Killiany Atlas. Automated parcellation results do not significantly differ from manual labeling reported by the FreeSurfer group [38], and there are no statistically significant changes in repeated measures of cortical thickness over several weeks in reproducibility tests [31]. Brain volumes were measured independently at two time points, so the FreeSurfer longitudinal procedure for vertex-based normalization was not required. An experienced neuroscientist blinded to clinical diagnosis visually inspected FreeSurfer pial and white borders and when necessary made minor manual corrections. RM-ANOVA was used to analyze effects of time by group interactions on volumes of cortical structures. Volumes at each time point were examined for group differences using univariate ANOVA. Within each group, measures at the two time points were compared using RM-ANOVA to identify changes over time. All volumetric analyses were conducted in SPSS, with age, gender and an average of intracranial volume (ICV) at both time points controlled.

### Demographics and symptom analyses

Survivors’ ages and the time intervals between MVC and the MRI sessions were compared for the two groups using two-sample T tests in SPSS. RM-ANOVAs were used to analyze effects of time by group interactions on PCL scores. Group differences in PCL scores, acute pain scores, and MSPSS scores at each time point were examined using univariate ANOVAs. Partial correlation analysis between the extracted COPEs or brain volumes and PCL scores examined relationships between brain measures and PTSD symptom severity. Gender and age were controlled in all ANOVAs and correlation analyses. Brain measures are reported as mean ± standard error of the mean.

## Results

### Symptoms assessments

All 38 survivors completed the PCL at both time points with the exception of one subject who missed the initial PCL. Sixteen (6 male, 10 female) probable PTSD group survivors screened positive for full (n = 13) or partial (n = 3) PTSD 3 months after MVC. The remaining 22 survivors (6 male, 16 female) who did not meet criteria comprised the non-PTSD group.

The time by group interaction for PCL scores was not significant (F = 0.051, p = 0.82). However, the group effect on PCL scores was significant (F = 82.894, p<0.001), and post hoc analysis revealed that PCL scores were significantly higher at two weeks and three months in the probable PTSD group than the non-PTSD group (Table 1). Both groups reported high levels of acute pain on the NPS in the ED, but the scores were significantly higher in the probable PTSD group than the non-PTSD group (Table 1). Survivors had had traumatic experiences as they suffered MVCs involving moderate to severe vehicle damage, were transported to the ED for immediate medical treatment, and suffered from high levels of pain caused by the MVC.

**Table 1:** Demographics and symptoms of probable PTSD and non-PTSD groups.

	Probable PTSD^¥^	Non-PTSD^¥^	Test	P value
**Number of survivors**	16	22		
**Male/Female (m/f)**	6m/10f	6m/16f		
**Age (years)**	31.6±9.5	34.7±13.2	t = 0.84	0.40
**Post-MVC days of the initial scan**	11.1±4.9	8.8±4.2	t = 1.5	0.14
**Post-MVC days of the follow-up scan**	110.1±13.2	108.7±16.5	t = 2.7	0.79
NPS scores at ED*	8.1±1.9	6.8±2.3	F = 5.98	0.02
PCL scores within 2 weeks*	57.2±12.8	31.1±8.8	F = 51.4	<0.001
PCL Scores after 3 months*	49.8±10.6	24.0±7.8	F = 75.3	<0.001
**MSPSS scores**	20.5±8	19.6±7.9	F = 0.16	0.70
**Comorbidity (number of survivors)**	mTBI (5), MDD (2), GAD (3), OCD (1), Lifetime PTSD (1)	mTBI (10), OCD (2), Substance Dependence (1)		
**Psychotropic medications**^**#**^ **(number of survivors)**	Pain Medication (7), Prozac/Xanax (1), Trazadone (1)	Pain Medication (6), Valium (1), Celexa (1)		

¥: results are reported as mean ± standard deviation.

#: Comorbid conditions include mild traumatic brain injury (mTBI), generalized anxiety disorder (GAD), obsessive-compulsive disorder (OCD), and major depression disorder (MDD).

*: significant group differences at p<0.05 level.

Similar numbers of survivors in two groups were prescribed pain medications. Five probable PTSD survivors and ten non-PTSD survivors met mTBI diagnosis criteria. Thirty-three survivors completed MSPSS within a month after MVC. The two groups did not differ with respect to MSPSS scores, age, or time between MVC and initial or follow-up sessions. Thirty-six survivors completed M.I.N.I. interviews at three months. Comorbid psychiatric conditions and psychotropic medications were reported by survivors in each group (Table 1).

### Neural activation during appraisal within two weeks after MVC

**Fig 1:**
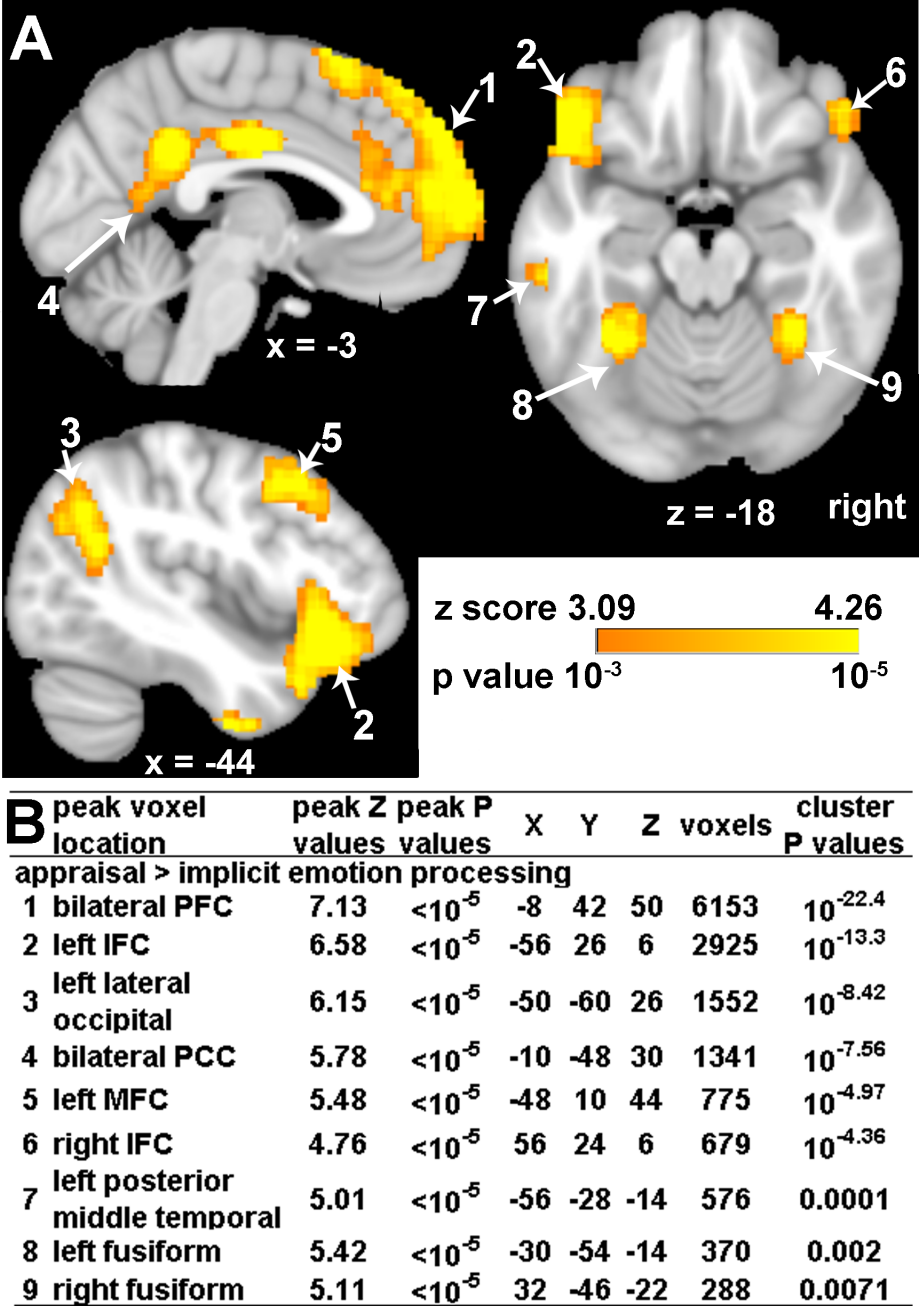
Main effect of appraisal of all emotional faces (fear, angry and neutral) as compared to implicit emotion processing. (A) A Z statistical map of significant clusters. The clusters are identified by number in the description below. (B) Summary of significant clusters for appraisal activation, initial cut-off voxel level Z > 3.09 (P < 0.001), with whole brain FWE correction at the cluster level of P<0.05.

Compared to implicit emotional processing, appraisal of all emotional faces was associated with greater bilateral activation in PFC, posterior cingulate (PCC), inferior frontal (IFC), and fusiform cortices, and greater left activation in lateral occipital, middle frontal (MFC), and posterior middle temporal cortices for all survivors at 2 weeks after MVC (Fig 1). As expected, the PFC cluster overlaps a previously reported left dmPFC (-12, 32, 58) active region seen in healthy people on this task [32]. The probable PTSD and non-PTSD groups did not differ in this contrast.

**Fig 2:**
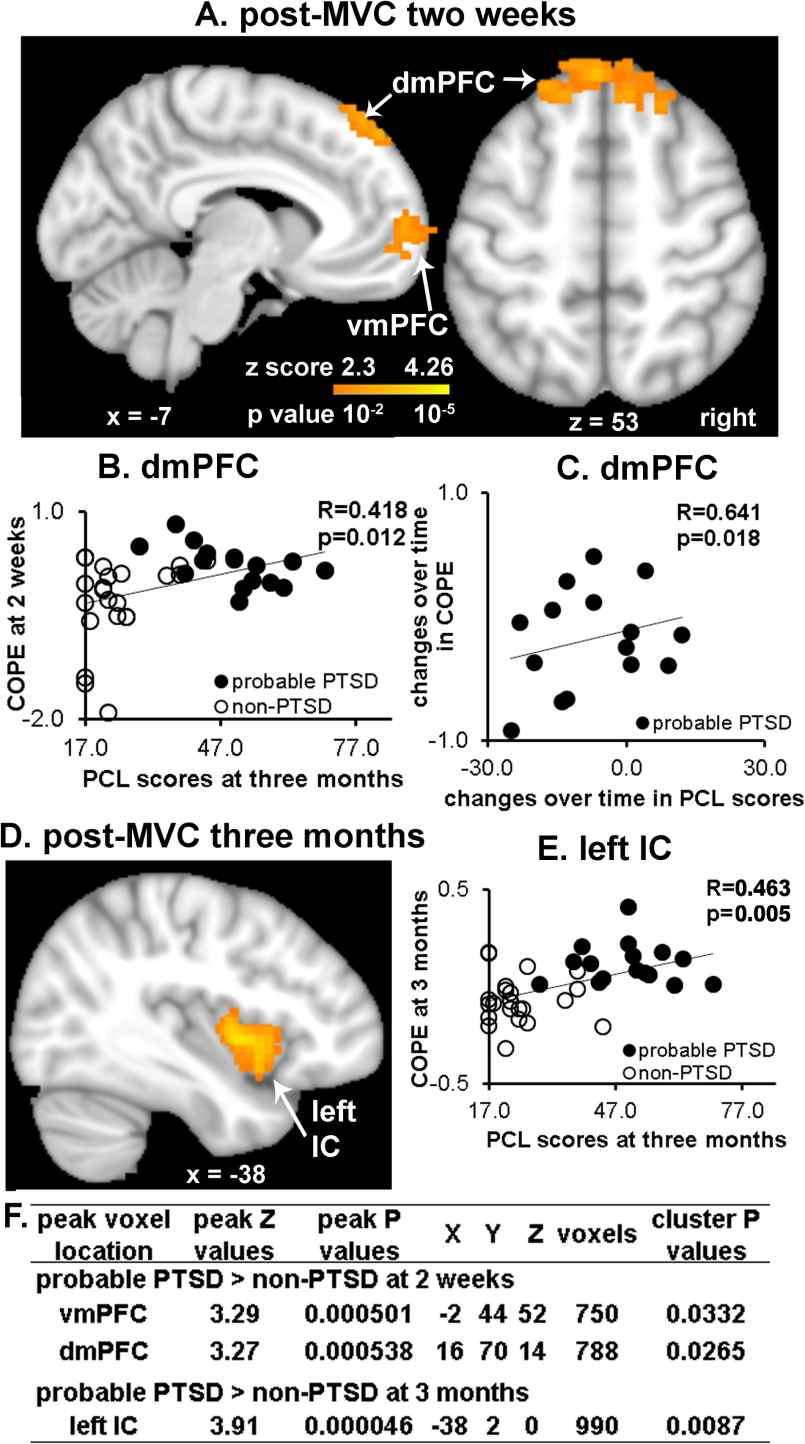
Differences in brain activation in response to fearful faces during appraisal tasks in probable PTSD and non-PTSD groups at two weeks and three months after MVC. (A) Appraisal of fearful faces vs. neutral faces reveals greater activation in dmPFC (sagittal and axial views) and vmPFC (sagittal view) in the probable PTSD group compared to the non-PTSD group at two weeks. (B) Activation in dmPFC at two weeks after MVC was significantly positively correlated with PCL scores at three months across both groups. (C) Changes over time in dmPFC activation and PCL scores from two weeks to three months were positively correlated in the probable PTSD group. (D) The same contrast reveals greater activation in the probable PTSD group compared to the non-PTSD group in left IC at three months. (E) Activation in left IC was positively correlated with PCL scores at three months across both groups. (F) Summary of significant differences in activation of appraisal of fearful faces between groups at both time points. Initial cut-off voxel level Z > 2.3 (P < 0.01), with whole brain FWE correction at the cluster level of P<0.05.

Whole brain voxel-based analysis of activation associated with appraisal of fearful vs. neutral faces revealed that activation to fearful faces was greater in dmPFC and vmPFC in the probable PTSD group than in the non-PTSD group (dmPFC: -2,44,52, z = 3.27; vmPFC: 16,70,14, z = 3.29; controlling for age and gender, FWE corrected; Fig 2A). COPE was extracted from the peak coordinates in dmPFC and vmPFC for further ROI analyses of task-related activations. After controlling for acute pain scores, extracted dmPFC COPEs remained significantly different between groups (probable PTSD: 0.21±0.07 vs. non-PTSD: -0.29±0.13; F = 10.621, P = 0.003; age, gender, and pain scores controlled), but extracted vmPFC COPEs were no longer significant (probable PTSD: -0.01±0.10 vs. non-PTSD: -0.13±0.16; F = 0.429, P = 0.52). dmPFC COPEs across all subjects at two weeks were positively correlated with PCL scores at both two weeks (R = 0.462, P = 0.006, df = 32) and three months (R = 0.418, P = 0.012, df = 33; Fig 2B) after MVC when age, gender, and pain scores were controlled. The early dmPFC and vmPFC COPEs were not significantly correlated with changes in PCL scores from two weeks to three months after MVC. Unlike fearful face results, whole brain voxel-based analysis revealed that the two groups did not differ in activations associated with appraising angry faces compared to neutral faces at two weeks.

### Progression of early differences in appraisal activation in survivors who did or did not develop PTSD at three months after MVC

To assess potential progressions of differences in early activation associated with appraising fearful faces over the 3 months following MVC, we extracted COPEs from the follow-up scans using the above peak coordinates in dmPFC and vmPFC. The change over time in extracted dmPFC COPEs in the probable PTSD group was significantly positively correlated with the change over time in PCL scores (R = 0.641, P = 0.018, df = 11; age, gender, and pain scores controlled; Fig 2C), but this correlation was not significant in the non-PTSD group (R = -0.13, P = 0.597, df = 17).

The time by group interaction was significant in extracted dmPFC COPEs (F = 7.941, p = 0.008; age, gender, and pain scores controlled), but changes over time in the extracted COPEs were not significant in either group (probable PTSD group: 0.21±0.07 to 0.12±0.11, F = 1.60, P = 0.23; non-PTSD group: -0.29±0.13 to 0.09±0.07, F = 0.87, P = 0.37; age, gender, and pain scores controlled). No significant findings were observed for extracted COPEs from the peak coordinate in vmPFC.

### Neural activation during appraisal three months after MVC

The whole brain voxel-based group comparison of activation associated with appraising fearful vs. neutral faces was repeated at 3 months after MVC. Compared to the non-PTSD group, the probable PTSD group had greater activation in left IC (-38, 2,0; z = 3.91, controlling for age and gender, FWE corrected; Fig 2D). Extracted COPEs from the peak coordinate in left IC positively correlated with PCL scores at three months after MVC across all subjects (R = 0.463, P = 0.005, df = 33, age, gender, and pain scores controlled; Fig 2E), but the correlations of changes over time in left IC and in PCL scores were not significant in either group. At three months, there was no difference in mPFC task-related activation between probable PTSD and non-PTSD groups in whole brain voxel-based analysis. The significant group differences in whole brain analyses at both time points were summarized in Fig 2F.

### Changes in cortical volume

**Fig 3:**
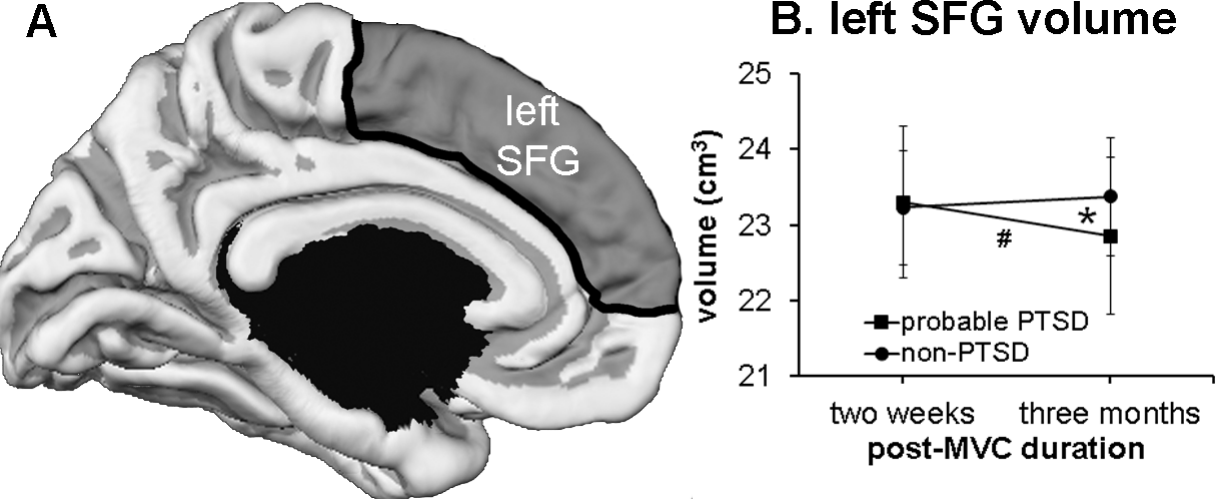
Volume of left SFG. (A) parcellation of left SFG (shaded in gray) in FreeSurfer partially overlaps with location of the dmPFC activation cluster. (B) Volume of left SFG at two weeks and three months after MVC. *: indicates significant effect of time by group interaction. #: indicates significant decrease in left SFG volume in probable PTSD group over time. The error bars indicate the standard error of the mean.

The dmPFC activation described above is located in the superior frontal gyrus (SFG) in both sides. We examined SFG structure by analyzing SFG volumes with RM-ANOVA. A significant time by group interaction (F = 6.048, p = 0.019) was found for left SFG volume (Fig 3). Left SFG volume significantly decreased from two weeks to three months in the probable PTSD group (23.3±1.0 cm^3^ to 22.9±1.0 cm^3^; F = 5.634, p = 0.035), whereas in the non-PTSD group the left SFG volume did not change during the same interval (23.2±0.8 cm^3^ and 23.4±0.8 cm^3^; F = 0.432, p = 0.519). Left SFG volumes were not significantly different between the two groups at either time point. The left SFG volumes at two weeks and changes over time in left SFG were not significantly correlated with changes over time in PCL scores. No significant group difference was found in right SFG volumes.

## Discussion

Chronic PTSD has been associated with altered activation in emotion circuits, but early effects of traumatic experiences on emotion activation that lead to PTSD development remain unknown. The present results suggest that by two weeks after MVC, dmPFC and vmPFC activations during appraisal of fearful faces were greater in the probable PTSD than non-PTSD group. The early dmPFC activation at two weeks positively correlated with PCL scores at three months, suggesting that acute dmPFC activation may be associated with subsequent PTSD symptom development. At three months, the differences in task-related activation in dmPFC and vmPFC in the two groups were no longer significant. However, by three months greater activation developed in left IC in the probable PTSD versus the non-PTSD survivors. Different activation patterns at these time points suggest different dynamic changes occur across emotional circuit locations during the initial weeks to months after MVC trauma. Moreover, changes over time in dmPFC activation were positively correlated with symptom progression in the probable PTSD group. Finally, progressive changes in volume of left SFG, which included the location of dmPFC activation, suggest a further contribution of structural changes to cortical activation changes that are related to PTSD symptoms.

### mPFC activation within two weeks after MVC

It has been reported that mPFC activation to negative emotional stimuli increases within 2 weeks after trauma exposure [17]. As shown by magnetoencephalography, greater prefrontal activity to aversive pictures was reported in PTSD subjects around 25 days after trauma [18]. Our findings suggest that early mPFC responses to negative stimuli are associated with PTSD symptoms in the months after trauma. mPFC is considered an emotion modulation region, and appraisal or reappraisal of negative emotional information activates vmPFC and dmPFC [39, 40]. Survivors who eventually develop PTSD may be more reactive to negative emotional stimuli than survivors who do not develop PTSD [41], and appraisal of fearful faces may, thus, lead to greater mPFC activation in these survivors. We surmise that greater early activation in dmPFC to fearful faces in survivors who develop PTSD symptoms may reflect compensatory dmPFC down-regulation of strong emotional responses. Consistent with this thinking, the greater early dmPFC activation in probable PTSD survivors is located in an active region identified during appraisal tasks in our previous report [32]. Furthermore, greater early dmPFC activation in probable PTSD survivors significantly positively correlates with PTSD symptom severity at 3 months. Fearful faces have been commonly used in emotion induction studies [42]. The findings obtained using fearful faces stimuli would likely generalize to fear evoking stimuli, however not necessarily other emotions. We did not find similar mPFC responses to angry faces in this study. Our previous study using SEAT suggested that angry facial expressions evoke different responses than fearful faces [43]. Furthermore, appraisal of positive emotion has not been studied. Thus, the present mPFC activation during appraisal of fearful faces may not generalize to different emotions, but this has to be tested empirically.

By contrast, we found less dmPFC activation in non-PTSD survivors compared to the probable PTSD group. Decreased mPFC activity has been reported when subjects suppress negative emotions [44]. Less activation in the acute posttraumatic period in non-PTSD survivors compared to the probable PTSD group may reflect successful suppression of negative emotional responses, whereas greater activation in the probable PTSD survivors may reflect failure of suppression of negative emotional responses and subsequent recruitment of other regulatory strategies like appraisal.

Finally, pain may affect activation in emotion-related regions including mPFC, and pain may negatively interact with emotional task-related activation in mPFC [45]. Acute group differences in vmPFC activation became insignificant after controlling for acute pain scores, suggesting that vmPFC activation may be influenced by pain. In contrast, controlling for acute pain scores did not affect the dmPFC findings, suggesting that acute dmPFC activation differences and their relationship to PTSD symptom development are less related to acute pain.

### dmPFC activation during PTSD development

In the probable PTSD group, dmPFC changes in appraisal activation over time positively correlated with the progression of PTSD symptoms. If dmPFC activation contributes to a compensatory emotion regulating process, this positive correlation suggests that progressive worsening of PTSD symptoms during the initial post-trauma period may prompt increases in dmPFC emotion regulation or vice versa.

### Left IC activation at three months after MVC

IC activation has been reported to be greater during emotional tasks in chronic PTSD patients than in non-PTSD survivors, and positively correlates with PTSD symptom severity [7] and heightened experience of aversive states [6, 7]. IC integrates feelings from the body and contributes to emotional awareness [46]. While, our findings confirm greater IC activation in PTSD survivors at 3 months, we did not find differences in IC activation between probable PTSD and non-PTSD survivors at two weeks after MVC. However, the negative findings in IC at 2 weeks require confirmation in larger samples. If confirmed, greater activation in IC associated with PTSD may develop over initial months following trauma.

### Reduced left SFG volume in probable PTSD survivors over initial three months after MVC

The probable PTSD survivors showed progressive left SFG volume decreases from two weeks to three months after MVC. Increasing evidence suggests that stress may lead to PFC neurochemical or substrate changes. These include, e.g., enhanced dopaminergic and noradrenergic receptor signal transduction, increased serotonin turnover, and/or reduction of neuronal dendrites [47, 48]. Neuroimaging studies suggest that traumatic experiences may rapidly alter the macro-structure of frontal cortical regions. Earthquake survivors exhibited reduced grey matter density in orbitofrontal cortex (OFC) 2–3 months after as compared to before the earthquake. Moreover, decreases in OFC grey matter density negatively correlated with posttraumatic stress levels [49]. We found partially overlapping emotion activation and volume changes in SFG, pointing to a potential relationship between progressive cortical activation and volume changes during PTSD development. Although about 30% of survivors in the probable PTSD group sustained mTBI during MVC, it is likely changes during 3 months in left SFG volume in this group were not attributed to mTBI because about 50% of survivors in the non-PTSD control group also had mTBI, but did not have left SFG volume changes over this time.

### Limitations

The current study has limitations that should be considered when interpreting the findings. By choosing to study MVC, a single type of trauma, we gained the advantage of trauma homogeneity but may limit generalizability of findings to other types of trauma. The present results are based on relatively limited sample sizes. Additionally, PTSD symptoms were assessed using the PCL self-report survey, but not structured interviews, such as Clinician Administered PTSD Scale (CAPS). While measuring the same 17 PTSD symptoms, PCL has different psychometric properties (sensitivity and specificity) than gold standard structured interviews like CAPS, and therefore we used the description of “probable PTSD”, rather than PTSD. Furthermore, although the current findings identify early brain activation differences across groups that preceded PTSD diagnosis at 3 months, these findings do not necessarily imply a causal effect between early brain changes and later PTSD symptoms. The significant positive correlation between PCL scores and dmPFC activations at 2 weeks suggests the possibility that early brain changes reflect early onset of PTSD symptoms, but this requires further attention. Finally, MVC survivors in both groups sustained physical injuries and acute pain. Given the current finding of different effects of pain on dmPFC and vmPFC, further study of the effects of acute pain on the brain mechanisms of PTSD development is needed.

## Conclusions

This is one of the few prospective studies to examine early and progressive brain changes that may underlie development of PTSD symptoms following a traumatic event. Our findings of cortical activation and volume differences in probable PTSD and non-PTSD survivors suggest potential cortical functional and structural mechanisms for development of PTSD symptoms. Further longitudinal studies on early brain changes may provide a basis for future interventions to prevent or reduce development of PTSD symptoms after trauma and for biomarker identification to evaluate clinical interventions.
